# Accuracy of Hepatobiliary Scintigraphy after Liver Transplantation and Liver Resection

**DOI:** 10.1155/2016/7857849

**Published:** 2016-08-03

**Authors:** Manuel Eckenschwiller, Hanns Ackermann, Wolf O. Bechstein, Frank Grünwald

**Affiliations:** ^1^Department of Nuclear Medicine, Goethe University, 60590 Frankfurt, Germany; ^2^Department of Biostatistics, Goethe University, 60590 Frankfurt, Germany; ^3^Department of General and Visceral Surgery, Goethe University, 60590 Frankfurt, Germany

## Abstract

*Background and Aims. *Biliary complications are the most frequent complications after common liver surgeries. In this study, accuracy of hepatobiliary scintigraphy (HBS) and impact of hyperbilirubinemia were evaluated.* Methods. *Between November 2007 and February 2016, 131 patients underwent hepatobiliary scintigraphy after having liver surgery. 39 patients with 42 scans after LTX (*n* = 13) or hepatic resection (*n* = 26) were evaluated in the study; 27 were male, with mean age 60 years. The subjects underwent hepatobiliary scintigraphy with Tc-99m labeled Mebrofenin. The results were compared to ERCP as gold standard performed within one month after HBS. We calculated sensitivity, specificity, PPV, and NPV. We compared LTX patients to patients with other liver surgeries. Furthermore the influence of hyperbilirubinemia on HBS scans was evaluated.* Results. *HBS always provided the correct diagnosis in cases of bile leak in the liver-resected group (14/14). Overall diagnostic accuracy was 76% (19/25) in this group and 54% (7/13) in the LTX group. False negative (FN) diagnoses occurred more often among LTX patients (*p* = 0.011). Hyperbilirubinemia (>5 mg/dL) significantly influenced the excretion function of the liver, prolonging HBS's time-activity-curve (*p* = 0.001).* Conclusions. *Hepatobiliary scintigraphy is a reliable tool to detect biliary complications, but reduced accuracy must be considered after LTX.

## 1. Introduction

Hepatic resection is widely used for the curative treatment of primary and secondary malignant as well as benign liver diseases [1]. In case of liver failure and end-stage liver disease, liver transplantation is required [2, 3]. In spite of great improvements in surgical techniques, however, postoperative complications remain a major cause of mortality and morbidity. For LTX and other liver surgeries, biliary tract complications such as bile leak or strictures are the most frequent postoperative complications [1, 4, 5]. Therefore a diagnostic tool to detect these complications should preferably be easy to perform, be noninvasive, and provide reliable results. The current gold standard in detecting biliary abnormalities is endoscopic retrograde cholangiopancreatography (ERCP) which is an invasive but reliable method and also provides interventional opportunities [6].

Other noninvasive diagnostic modalities are used to detect biliary complications after common liver surgery but are mostly limited due to different factors. Ultrasound is a quickly applicable, cheap, and broadly available diagnostic tool; however, it does not provide reliable results on biliary strictures [7] and bilomas [8] and can therefore only be used as screening or supplementary tool. For the evaluation of biliary complications, X-ray computed tomography is inferior to ERCP and magnetic resonance cholangiography, although it is useful in detecting vascular problems, abscesses, and bilomas. For the sole assessment of biliary strictures, magnetic resonance cholangiography is considered a reliable and noninvasive method [8]. HBS scans are used as primary or supplementary diagnostic tools. Current study data evaluating the usefulness of hepatobiliary scintigraphies in liver transplanted patients are broad; however, most studies deal with specific details such as living donor liver transplantation [9, 10] or pediatric LTX [11]. Reliable studies that actually examined the role of HBS in liver-resected patients are rare [12], because most of those studies focused on preoperative evaluation of liver function [13, 14]. Only a few studies with strict and reasonable inclusion criteria and a prospective study design were performed, focusing on LTX patients [15]. There has not yet been a study investigating differences in the usefulness of HBS between liver transplanted and liver-resected patients. Moreover, the role of hyperbilirubinemia and its impact on cholescintigraphic scans remain unclear, as a study found bilirubin to have a negative influence on the diagnosis provided by HBS [16].

The aim of this study was to determine the usefulness of hepatobiliary scintigraphy in diagnosing the most frequent biliary complications after common liver surgery and to evaluate the influence of bilirubin on hepatobiliary scintigraphy scans.

## 2. Materials and Methods

This retrospective study was approved by the ethical review committee of the J. W. Goethe University Medical Center in Frankfurt, Germany (reference number: 27/13), and the need for written informed consent was waived.

### 2.1. Clinical Setting and Patients

Between November 2007 and February 2016, 131 patients underwent hepatobiliary scintigraphy after performed liver surgery (transplantation or resection). Inclusion criteria were an ERCP within one month (31 days) after the HBS and, furthermore, blood analyses performed within a maximum time of 2 days from the HBS. 39 patients with *n* = 42 hepatobiliary scintigraphies (3 patients were examined twice) met inclusion criteria. 26 (66.7%) had undergone partial liver resection (hemihepatectomy, partial hepatectomy, or cholecystectomy) and 13 were liver transplanted patients. 27 (69%) were male and the mean age was 60 years.

### 2.2. Technical Equipment and Pharmaceuticals

#### 2.2.1. Tracer

Mebrofenin (Bridatec, GE Healthcare Buchler GmbH & Co. KG, Braunschweig, Germany), labeled with radioactive Tc-99m, was used as the tracer (1.11 MBq per kg body weight). The effective equivalent dose of Tc-99m is 0.017 mSv per MBq resulting in a total of 1.36 mSv for one examination of a patient weighing 70 kg. The maximum activity for one patient was 148 MBq.

#### 2.2.2. Gamma Camera

Images were acquired with a large-field double-head gamma camera (DST-Xli, GE Healthcare, Solingen, Germany) equipped with a low-energy high resolution (LEHR) collimator.

#### 2.2.3. Documentation

Documentation of the images was done using RIS/PACS software (GE Healthcare, Dornstadt, Germany) and the analysis was performed using Xeleris software, version 3.0423 (GE Healthcare, Dornstadt, Germany).

### 2.3. Measurement and Evaluation

#### 2.3.1. Scintigraphy

To avoid gall bladder contraction, the patients had to fast and were not allowed to smoke at least two hours prior to the examination. After intravenous bolus injection of Tc-99m-Mebrofenin with 1.11 MBq per kg body weight, dynamic sequences were started simultaneously from anterior and posterior views. To evaluate the perfusion phase, 60 frames were obtained with time intervals of one second each at the Tc-99m peak of 140 KeV with a 20% window, a 128 × 128 matrix (256 × 256 for static images), and a zoom of 1.33. After this first minute, another dynamic sequence was run with 30 frames and time intervals of 120 seconds each, taking 61 minutes for a complete dynamic acquisition. Static images were then obtained from anterior, posterior, and, if necessary, oblique and lateral views. They were taken 70, 80, and 90 minutes after injection for 10 minutes each. If abdominal activity was elevated and a maximum of 600,000 counts was reached before the time of 10 minutes, the acquisition was stopped earlier. If the patient lacked parenchymal clearance, more static images were taken until 24 hours after injection.

#### 2.3.2. Evaluation of Hepatobiliary Scintigraphy

**Figure 1:**
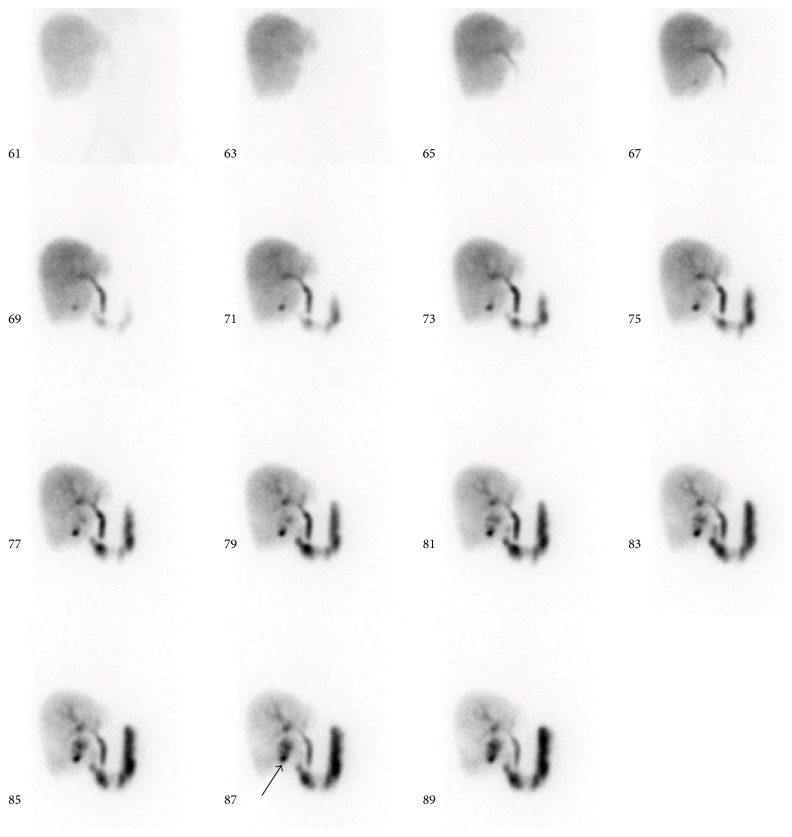
Bile leak (arrow) in a patient after partial liver resection and cholecystectomy (parenchymal phase).

**Figure 2:**
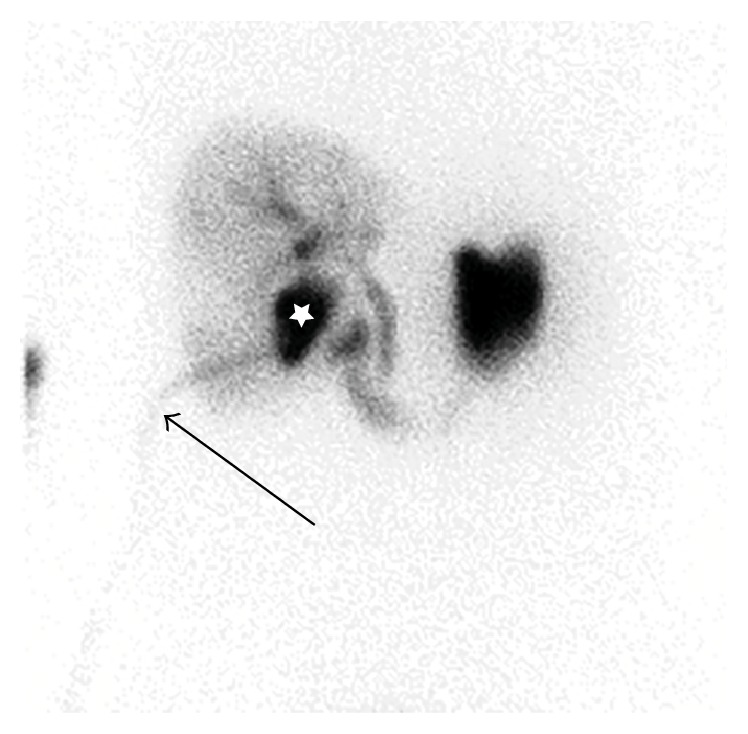
Bile leak (white star) in a liver and gallbladder-resected patient and bile transportation over the drainage (arrow).

Quantitative evaluation was performed using Xeleris software, version 3.0423 by combining the dynamic images of the last 30 frames (120 sec/each) to make one sum picture. Two or three regions of interest (ROI) were then placed in this image: one over the liver in a noncentral part (to avoid measuring bile ducts), one over the duodenum, and, if necessary, one over the drainage bag. With the resulting time-activity curves (TAC), evaluation of liver uptake, excretion function, and transport to the bowel were performed and the peak of each curve was determined. The peaks were then compared to standard values: 10 ± 3 min for the uptake maximum in the liver and 20 ± 8 min in the duodenum. After 60 minutes, the liver parenchyma was expected to be nearly activity-free. Using these criteria, it was possible to analyze the parenchymal function and the bile excretion and transport into the duodenum. Possible diagnoses were bile leak, obstruction, and parenchymal dysfunction. All results were reviewed by experienced physicians. Parenchymal dysfunction was diagnosed if the uptake maximum appeared after more than 13 minutes after injection representing a prolonged clearance of the liver. Bile leak was diagnosed if tracer accumulated at a nonphysiological abdominal site (Figures 1 and 2). The diagnosis of biliary obstruction was made if the dynamic images showed a prolongation of the transport to the bowel or if bile retention was observed in the static images.

#### 2.3.3. ERCP

ERCP was performed at our institutional gastroenterologic department as the gold standard for our study within a maximum time of 31 days after the HBS. With this short period of time, we tried to avoid incorrect diagnoses due to changes in the biliary system. Because this was a retrospective study and ERCP was performed after the HBS, the physicians performing the HBS were blinded automatically. The ERCP was performed by at least one senior physician of the gastroenterologic department with one resident or another senior physician. Possible diagnoses were bile leak or obstruction and there was the possibility to extend the bile ducts with a balloon and implant stents.

#### 2.3.4. Blood Parameters

Blood test analysis was performed at our institutional laboratory prior to the HBS scan, when possible, on the day of the examination. If lab data were not available on the same day, we took the closest results to the HBS with a maximum time of two days between blood sampling and scintigraphy.

#### 2.3.5. Evaluation of Results

Hepatobiliary scintigraphy results were compared to ERCP as the gold standard and were categorized as true positive (TP), true negative (TN), false positive (FP), false negative (FN), and inconclusive. Results were considered positive when the HBS provided a nonphysiological result (e.g., biliary obstruction) that later was confirmed by ERCP (TP) or not confirmed (FP). Negatives were considered all results from the HBS that did not show abnormal liver function or tracer accumulation. These results were also compared to ERCP and were either confirmed (TN) or negated (FN). Using these values, we calculated sensitivity and specificity as well as positive predictive value (PPV) and negative predictive value (NPV). We compared patients with liver transplantation (LTX) to patients who had other types of surgery (hemihepatectomy, partial resection, or cholecystectomy). We also compared patients with elevated total bilirubin to patients who had normal bilirubin values. We calculated results for both 1 mg/dL and for 5 mg/dL as the upper thresholds of the standard total bilirubin values. At our institution 1 mg/dL was the standard bilirubin value, but since 5 mg/dL has shown to be statistically significant in other studies, we used both. Patients were categorized into three groups according to the peak of their TAC: 0–13 minutes was considered a normal excretion function of the liver; 14–20 minutes was categorized as moderate prolongation, and more than 20 minutes was considered severe prolongation. These three groups were analyzed for statistically significant differences due to elevated bilirubin levels. To evaluate the differences regarding the usefulness of the HBS scans, we also compared patients who were diagnosed by ERCP to have either bile leak, obstruction, or no findings. We analyzed whether there were any statistical differences with respect to blood tests, sensitivity, specificity, PPV, and NPV.

### 2.4. Statistical Analysis

Statistical analysis was performed using BiAS, version 10.03 for Windows (epsilon-Verlag, Hochheim Darmstadt, Germany). Data were analyzed using the chi^2^-test to compare the group of LTX patients with the group of liver-resected patients in relation to the diagnosis (TP, TN, FP, and FN). It was also used to analyze the dependence of the TAC on total bilirubin levels. For a more specific evaluation of the results of the chi^2^ test, we used Fisher's exact test to determine which diagnosis was influenced most significantly. Kruskal-Wallis' test was used to determine whether there was a significant distribution of quantitative values, such as blood tests, between the study groups. Results were considered statistically significant for a *p* value <0.05.

## 3. Results

Table 1 shows the characteristics of the study subjects.

Table 2 shows the median and the range of the blood tests of the study subjects at the time of the HBS scan. Kruskal-Wallis' test showed that those values associated with the liver such as hepatic enzymes, bilirubin, albumin, or coagulation parameters were not statistically significant distributed between the final diagnoses (TP, TN, FP, and FN). The associated *p* values between the laboratory values and the diagnoses are shown in Table 2.

The comparison of LTX patients to patients who underwent liver resection showed a statistically significant difference in the distribution of results between both groups (Table 3) with a *p* value = 0.035. This was due to the FN diagnoses showing a significant distribution with a *p* value = 0.011. The reason was the group of LTX patients in which 6 of 13 scans (46%) were FN while in the resection group; there were only 2 FN out of 25 (8%). TP (*p* = 0.495), TN (*p* = 1.0), and FP (*p* = 0.278) were not distributed significantly between the two groups.

26 out of 42 scans were read correctly and 12 were read falsely. Four patients included in the study were classified inconclusive regarding the diagnoses (TP, TN, FP, and FN): 2 patients were diagnosed by the HBS to have biliary obstruction while ERCP showed bile leak. So, those diagnoses were neither right nor wrong and could therefore not be classified TP or FN. 2 patients were classified inconclusive because ERCP had to be cancelled due to intestinal edema in one case and intestinal food rests in the other. Data from those 4 patients were only evaluated in the second part of the study in which we studied the dependence of the TAC on bilirubin. There were differences between the two groups with respect to sensitivity, specificity, PPV, and NPV which are shown in Table 4.

For a more specific evaluation, we evaluated data from the two groups with respect to differences between the diagnoses, “obstruction,” “bile leak,” or “no findings” (Table 5), confirmed by ERCP. Of note were the 14 liver-resected patients with a “bile leak” determined by ERCP who all were diagnosed correctly (TP) by the HBS, leading to a sensitivity of 100% for the “bile leak” diagnosis in liver-resected patients. The HBS provided the correct diagnosis in 76% of cases (19/25) for resected patients while the diagnostic accuracy for LTX patients was 54% (7/13). The diagnostic accuracy of the leakage and obstruction in liver transplantation and resection is also shown in Table 5, as it is equal to the sensitivity here. Inconclusive cases were not used for calculation and are therefore not included in Table 5.

### 3.1. Impact of Hyperbilirubinemia on HBS Scans

The comparison of both groups regarding the impact of total bilirubin on the diagnosis (TP, TN, FP, and FN) did not show any statistical significance. Neither 5 mg/dL nor 1 mg/dL as the threshold value had any significant impact on the correctness of the HBS results and there were no significant differences between the two groups.

**Figure 3:**
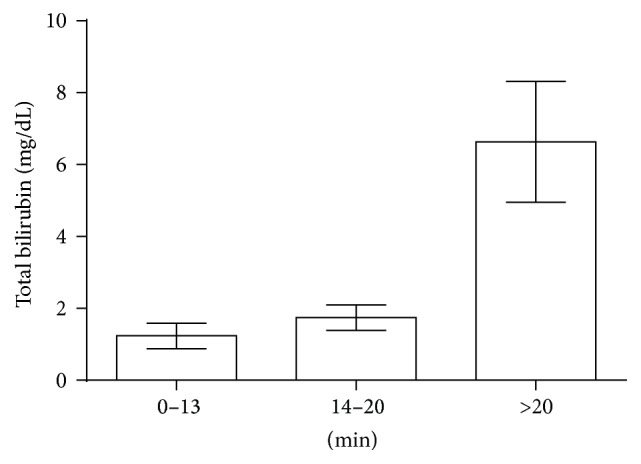
Mean bilirubin values and standard deviation (*y*-axis) in the three groups of TAC peaks (*x*-axis). mg: milligram; dL: decilitre; min: minutes.

**Figure 4:**
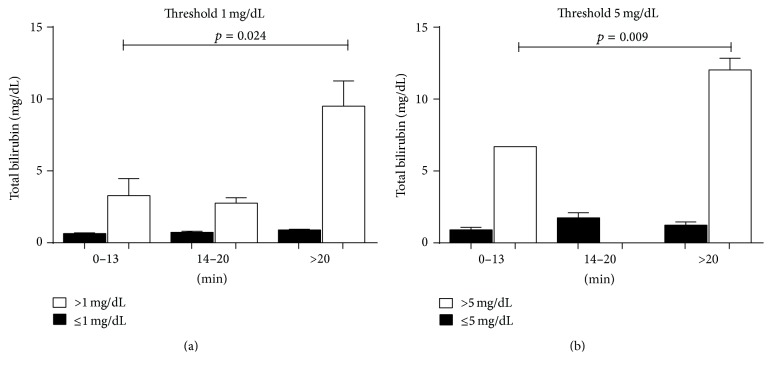
Peak of the TAC in the three groups (*x*-axis) in dependence on mean bilirubin levels plus standard deviations. The* p* values indicate a significant distribution of high bilirubin levels between the first (left) and the third (right) group. mg: milligram; dL: decilitre; min: minutes.

We found that the hepatic uptake of Mebrofenin, which is expressed through the TAC, is slowed down by high bilirubin levels.  Figure 3 shows the mean bilirubin levels in the three groups of TAC peaks with standard deviation (SD). The chi^2^-test showed a statistically significant dependence of those TACs on total bilirubin levels with a *p* value = 0.001 for a threshold of 5 mg/dL and *p* = 0.046 for a threshold of 1 mg/dL. Comparing the group with normal excretion function (peak of the TAC between 0 and 13 minutes) with the one with severe prolongation of excretion (>20 minutes), we found statistical significance for both thresholds (Figures 4(a) and 4(b)). When we compared patients with normal excretion function to all patients with prolonged excretion (>13 minutes), *p* was 0.029 for 1 mg/dL.

## 4. Discussion

### 4.1. Synopsis

We tried to evaluate the clinical usefulness of hepatobiliary scintigraphies compared to ERCP as the gold standard in patients who underwent common liver surgery, focusing on the comparison of LTX patients to liver-resected patients and on the HBS's dependence on total bilirubin levels. Our results showed statistically significant differences in finding the correct diagnosis between LTX patients and the group of liver-resected patients with a *p* value = 0.035. Dependence of the diagnosis on elevated total bilirubin could not be observed; however, we found that high levels of total bilirubin delayed the peak of the TAC of the HBS. Our study showed a remarkable difference between LTX patients and liver-resected patients with respect to the usefulness of the HBS.

### 4.2. Interpretation

Our study showed a significant difference between LTX patients and liver-resected patients, with respect to the diagnosis as well as remarkable differences in sensitivity, specificity, PPV, and NPV.

The relatively large amount of cases diagnosed as FN in the LTX group led to the significant difference between the two groups, to a low sensitivity (45%) and an even lower NPV (25%) for LTX patients. Other studies showed sensitivity ranging between 50% and 100% [3, 9, 10, 15]. This large range is most likely due to different study performances, different measurements, and other evaluation methods. In 1997, Kurzawinski et al. [15] performed a prospective study with LTX patients only, using similar inclusion criteria and study design as we did. Their results were similar to the now presented data, only their specificity for biliary complications was lower than in our study [15]. A possible explanation for the low sensitivity and accuracy of the HBS in LTX patients might be due to the development of complications after performed hepatobiliary scintigraphy. We tried to rule out these FN diagnoses with a short period (1 month) between the HBS and ERCP, but it is nevertheless possible that changes might have occurred rendering the results different from the HBS. LTX being a more invasive and complex procedure as partial liver or gallbladder resection might lead to a broader variety of complications that are more difficult to detect by scintigraphy. This might explain why the HBS did not give reliable results for LTX patients who actually had complications.

The results for detection of obstruction in the liver-resected group were not as good as in other studies [12]. Most comparable, retrospective studies showed good results for the detection of bile leak [3, 12, 16], as did ours. This seems likely as bile leaks are easy to detect by scintigraphy, for example, tracer accumulation in a nonphysiological site as shown in Figure 2.

Kurzawinski et al. calculated a low sensitivity (50%) for bile leak detection in their study [15]. Their results indicate that the HBS is more useful in patients who have undergone liver or gallbladder resection than in LTX patients. The FP diagnoses in liver-resected patients affect PPV (78%) and specificity (56%) but usually have no bad influence on the treatment, as the patient receives further examinations to confirm the diagnosis. Our results show that the hepatobiliary scintigraphy is an appropriate method to detect biliary complications in general.

Another study showed significant negative influence of hyperbilirubinemia (>5 mg/dL) on the diagnosis provided by the HBS in patients who underwent orthotopic liver transplantation [16]. A correlation between elevated total bilirubin and the final diagnosis could be observed in neither LTX patients nor in liver-resected patients. Yet there was a strong correlation between elevated total bilirubin and a prolonged peak of the TAC. This seems likely, as Mebrofenin is carried by the same transporters as bilirubin [17] and therefore the Mebrofenin uptake is competitively inhibited. Our results indicate that bilirubin > 5 mg/dL is a reliable cut-off-value (*p* = 0.001). Other lab values (Table 2) did not interact with the final diagnosis so that it can be concluded that alterations of those values do not influence the result of the HBS figures.

### 4.3. Relevance

Hepatobiliary scintigraphies are used to detect complications as obstructions or bile leaks especially in patients who have undergone common liver surgery. Low sensitivity and NPV show that, in our study, the HBS did not prove to be successful in detecting complications in LTX patients. Although specificity and PPV were 100% each, it is the FN diagnoses that play an important role in clinical practice. Being diagnosed negatively for biliary complications by the HBS affects further treatment and, in case of FN diagnoses, might delay necessary treatment of existing complications. For patients with liver or gallbladder resection, the HBS is an appropriate method in the postoperative evaluation of complications.

### 4.4. Limitations

Our study was retrospective, limiting the relevance of this study as it is more vulnerable to biases as a prospective study. We tried to minimize these biases with strict inclusion criteria. We focused solely on patients who had undergone ERCP as the gold standard within one month after the HBS to minimize the risk of severe intestinal changes leading to different diagnoses [15]. There were no interventions between the HBS and ERCP that could have changed the structure of the anastomosis or intestinal anatomy. However, it was impossible to completely rule out changes that could lead to false diagnoses and therefore to low sensitivity, specificity, PPV, and NPV. Due to our small sample size (42 scans; 39 patients), the validity of our study is limited. However, our inclusion and exclusion criteria were strict, so in spite of the small number of study subjects, the data resulting from this study are more reliable.

## 5. Conclusions

Hepatobiliary scintigraphy in general is an appropriate method to detect biliary complications after common liver surgery. However, as the HBS proved to be less sensitive in LTX patients, a negative result in a clinically suspicious patient should be confirmed by other diagnostic tests, preferably ERCP. HBS is a reliable method to detect bile leak, especially in liver-resected patients, and may therefore be used in the follow-up of these patients. Hyperbilirubinemia prolongs the hepatic uptake of Mebrofenin and prolongs the HBS's TAC, but the final result, the diagnosis, is not influenced. Therefore, hepatobiliary scintigraphy can also be performed in patients with high bilirubin levels. We recommend a restricted use of the HBS in LTX patients but advise this diagnostic tool to rule out bile leaks after liver surgery.

## Figures and Tables

**Table 1:** Characteristics of the study population.

Variable	Value
Age (years) median [range]	61 [34–81]
Male *n* [%]	27 [69]
Time surg. to HBS (days) median [range]	15.5 [0–4400]
Time HBS to ERCP (days) median [range]	7 [0–31]
LTX *n* [%]	13 [33]
Hemihepatectomy *n* [%]	7 [18]
Partial liver resection *n* [%]	11 [28]
Cholecystectomy *n* [%]	5 [13]
Resection + cholecystectomy combined *n* [%]	3 [8]

Surg. = surgery; HBS = hepatobiliary scintigraphy; ERCP = endoscopic retrograde cholangiopancreaticography; LTX = liver transplantation.

**Table 2:** Laboratory values versus HBS diagnosis (*p* values).

Lab value	Median [range]	Standard [unit]^*∗*^	*p* value
GOT (AST)	47 [17–130]	<40 [U/L]	0.658
GPT (ALT)	45 [8–255]	<50 [U/L]	0.661
GGT	259 [39–2010]	<60 [U/L]	0.372
Total bilirubin	0.9 [0.4–15.0]	<1 [mg/dL]	0.578
Alk. phosphatase	185 [62–1474]	40–130 [U/L]	0.681
Albumin	3.3 [1.9–4.8]	3.5–5.2 [g/dL]	0.269
PT	82 [43–122]	70–130 [%]	0.257
INR	1.1 [0.89–1.87]		0.297
PTT	36 [27–52]	25–37 [sec]	0.824

Lab = laboratory; GOT = glutamic-oxaloacetic-transaminase; GPT = glutamic-pyruvic-transaminase; GGT = gamma-glutamyl-transferase; alk. = alkaline; PT = prothrombin time; INR = international normalized ratio; PTT = partial prothrombin time; U = unit; L = litre; mg = milligram; dL = decilitre; sec = second.

**Table 3:** Diagnoses for all patients (overall) and differentiated by LTX and resection.

Procedure	Scans [%]	TP [%]	TN [%]	FP [%]	FN [%]	Inc. [%]	*p* value
*Overall*	*42 *[*100*]	*19 *[*45*]	*7 *[*17*]	*4 *[*9.5*]	*8 *[*19*]	*4 *[*9.5*]	*0.035*
LTX	14 [33]	5 [36]	2 [14]	0 [0]	6 [43]	1 [7]	
Resection	28 [67]	14 [50]	5 [18]	4 [14]	2 [7]	3 [11]	

LTX = liver transplantation; TP = true positive; TN = true negative; FP = false positive; FN = false negative; inc. = inconclusive.

**Table 4:** Accuracy values for all patients (overall) and differentiated by LTX and resection.

Procedure	Sensitivity (%)	Specificity (%)	PPV (%)	NPV (%)
*Overall*	*70*	*64*	*83*	*47*
LTX	45	100	100	25
Resection	88	56	78	71

LTX = liver transplantation; PPV = positive predictive value; NPV = negative predictive value.

**Table 5:** Diagnosis-dependent differences between LTX and liver-resected patients.

Procedure	*n* [%]	TP [%]	TN [%]	FP [%]	FN [%]	Sens. (%)	Spec. (%)	Acc. (%)
*LTX*	*13 *[*100*]	*5 *[*39*]	*2 *[*15*]	*0 *[*0*]	*6 *[*46*]	*45*	*100*	
Obstruction	6 [46]	3 [50]			3 [50]	50		50
Leak	5 [39]	2 [40]			3 [60]	40		40
No findings	2 [15]		2 [100]	0 [0]				

*Resection*	*25 *[*100*]	*14 *[*56*]	*5 *[*20*]	*4 *[*16*]	*2 *[*8*]	*88*	*56*	
Obstruction	2 [8]	0 [0]			2 [100]	0		0
Leak	14 [56]	14 [100]			0 [0]	100		100
No findings	9 [36]		5 [56]	4 [44]				

LTX = liver transplantation; TP = true positive; TN = true negative; FP = false positive; FN = false negative; sens. = sensitivity; spec. = specificity; acc. = diagnostic accuracy.

## References

[1] Jarnagin W. R., Gonen M., Fong Y. (2002). Improvement in perioperative outcome after hepatic resection: analysis of 1,803 consecutive cases over the past decade. *Annals of Surgery*.

[2] Moon D.-B., Lee S.-G. (2009). Liver transplantation. *Gut and Liver*.

[3] Al Sofayan M. S., Ibrahim A., Helmy A., Al Saghier M. I., Al Sebayel M. I., Abozied M. M. (2009). Nuclear imaging of the liver: is there a diagnostic role of HIDA in posttransplantation?. *Transplantation Proceedings*.

[4] Mueller A. R., Platz K.-P., Kremer B. (2004). Early postoperative complications following liver transplantation. *Best Practice and Research: Clinical Gastroenterology*.

[5] Wojcicki M., Milkiewicz P., Silva M. (2008). Biliary tract complications after liver transplantation: a review. *Digestive Surgery*.

[6] Sanna C., Giordanino C., Giono I. (2011). Safety and efficacy of endoscopic retrograde cholangiopancreatography in patients with post-liver transplant biliary complications: results of a cohort study with long-term follow-up. *Gut and Liver*.

[7] Beswick D. M., Miraglia R., Caruso S. (2012). The role of ultrasound and magnetic resonance cholangiopancreatography for the diagnosis of biliary stricture after liver transplantation. *European Journal of Radiology*.

[8] Singh A. K., Nachiappan A. C., Verma H. A. (2010). Postoperative imaging in liver transplantation: what radiologists should know. *Radiographics*.

[9] Kim J., Moon D., Lee S. (2002). The usefulness of hepatobiliary scintigraphy in the diagnosis of complications after adult-to-adult living donor liver transplantation. *European Journal of Nuclear Medicine*.

[10] Kim Y. J., Lee K. T., Jo Y. C. (2011). Hepatobiliary scintigraphy for detecting biliary strictures after living donor liver transplantation. *World Journal of Gastroenterology*.

[11] Concannon R. C., Howman-Giles R., Shun A., Stormon M. O. (2009). Hepatobiliary scintigraphy for the assessment of biliary strictures after pediatric liver transplantation. *Pediatric Transplantation*.

[12] Kim J. S., Moon D. H., Lee S. G. (2000). Hepatobiliary scintigraphy in the assessment of biliary obstruction after hepatic resection with biliary-enteric anastomosis. *European Journal of Nuclear Medicine*.

[13] de Graaf W., van Lienden K. P., Dinant S. (2010). Assessment of future remnant liver function using hepatobiliary scintigraphy in patients undergoing major liver resection. *Journal of Gastrointestinal Surgery*.

[14] Bennink R. J., Dinant S., Erdogan D. (2004). Preoperative assessment of postoperative remnant liver function using hepatobiliary scintigraphy. *Journal of Nuclear Medicine*.

[15] Kurzawinski T. R., Selves L., Farouk M. (1997). Prospective study of hepatobiliary scintigraphy and endoscopic cholangiography for the detection of early biliary complications after orthotopic liver transplantation. *British Journal of Surgery*.

[16] Hopkins L. O., Feyssa E., Parsikia A. (2011). Tc-99m-BrIDA hepatobiliary (HIDA) scan has a low sensitivity for detecting biliary complications after orthotopic liver transplantation in patients with hyperbilirubinemia. *Annals of Nuclear Medicine*.

[17] de Graaf W., Häusler S., Heger M. (2011). Transporters involved in the hepatic uptake of 99mTc-mebrofenin and indocyanine green. *Journal of Hepatology*.

